# A Preprocessing Method for Insulation Pull Rod Defect Dataset Based on the YOLOv5s Object Detection Network

**DOI:** 10.3390/s25041209

**Published:** 2025-02-17

**Authors:** Xuetong Li, Meng Cong, Bo Liu, Xianhao Fan, Weiqi Qin, Fangwei Liang, Chuanyang Li, Jinliang He

**Affiliations:** 1 Department of Electrical Engineering, Tsinghua University, Beijing 100084, China; lixt23@mails.tsinghua.edu.cn (X.L.); xianhaofan@tsinghua.edu.cn (X.F.); qinwq@mail.tsinghua.edu.cn (W.Q.); liangfangwei@tsinghua.edu.cn (F.L.); hejl@tsinghua.edu.cn (J.H.); 2 Shandong Taikai Electrical Insulation Co., Ltd., Taian 271000, China; congm@sdtaikai.com (M.C.); liubo@sdtaikai.com (B.L.)

**Keywords:** pull rod, defect detection, YOLOv5s, copy–paste, bounding box, Mosaic, data augmentation

## Abstract

Insulation pull rods used in gas-insulated switchgear (GIS) inevitably contain the micro defects generated during production. The intelligent identification method, which requires large datasets with a balanced distribution of defect types, is regarded as the prevailing way to avoid insulation faults. However, the number of defective pull rods is limited, and the occurrence of different types of defects is highly imbalanced in actual production, leading to poor recognition performance. Thus, this work proposes a data preprocessing method for the insulation pull rod defect feature dataset. In this work, the YOLOv5s algorithm is used to detect defects in insulation pull rod images, creating a dataset with five defect categories. Two preprocessing methods for impurities and bubbles are introduced, including copy–paste within images and bounding box corrections for hair-like impurities. The results show that these two methods can specifically enhance small-sized defect targets while maintaining the detection performance for other types of targets. In contrast, the proposed method integrates copy–paste within images with Mosaic data augmentation and corrects bounding boxes for hair-like impurities significantly improving the model’s performance.

## 1. Introduction

Gas-insulated switchgear (GIS) has been widely used in power grids [1,2], in which the insulation pull rod is employed as a key insulating component in GIS and is responsible for transmitting mechanical motion. Insulation pull rods are typically made from fiber-reinforced epoxy composites, and defects can occur during production, transportation, and installation. The presence of defects may lead to insulation breakdown and further affect the reliable operation of GIS equipment [3,4]. Therefore, accurate detection of insulation pull rod defects is essential to reduce the operational risks of GIS devices and ensure the high reliability of power grid operations.

In recent years, with the rapid development of artificial intelligence and deep learning technologies, deep learning-based object detection techniques have made significant progress. The YOLO (You Only Look Once) series is a typical single-stage detector widely used for object detection. In the power industry, YOLO-based detectors have been applied in various scenarios. Feng et al. [5] introduced the K-means clustering method into the YOLOv5x model to achieve automated detection of insulator defects in power transmission lines. Liu et al. [6] incorporated Ghost convolution and model pruning into the YOLOv5s network to reduce model parameters. This approach significantly improved detection speed without compromising accuracy and was used to detect whether safety harnesses were properly worn during high-altitude operations by power workers. Nguyen [7] proposed using AI-powered drones to automate the inspection process of power facilities. Based on the YOLOv5s network, the system monitored the condition of five types of electrical equipment: crossarms, isolators, insulators, poles, and transformers. Pérez-Aguilar et al. [8] developed an automated computer vision mechanism based on YOLOv5 to detect hot spots in thermal images of electrical substations. Li et al. [9] introduced an image enhancement method based on illumination correction and compensation to overcome lighting challenges. Combined with the YOLOv5 model, this method was used to detect insulator defects. Liang et al. [10] proposed a lightweight non-destructive testing algorithm for welding defect surfaces based on attention mechanism, which solves the problem of it being difficult to efficiently and accurately detect complex defects in the ultrasonic welding process. However, current research mainly focuses on the recognition of large targets, such as insulators and human bodies, and the detection accuracy still needs to be improved when dealing with small-sized defects. The insulation rod defects that need to be detected in this study are generally small-sized defects, and these targeted detection methods need to be studied.

Another study by Li et al. [11] leveraged the YOLOv5s model to detect typical defects in insulation pull rods. They demonstrated that YOLOv5s has a faster inference speed and higher recognition accuracy compared to other mainstream recognition models, including Faster R-CNN, Mask R-CNN, SSD, YOLOv4, etc., in identifying insulation rod defects. However, the dataset primarily consisted of insulation pull rods with highly distinctive features, resulting in decreased detection accuracy for rods with less prominent features encountered in real production procedures.

In practical applications, to maximize the accuracy and efficiency of a model, it is essential to ensure a large and diverse dataset. However, for defect detection in insulation pull rods, directly collecting a large number of images of typical defects is labor-intensive and incurs high production costs. Even if this data collection is feasible, the dataset may still be insufficient for model training. Therefore, data augmentation techniques are required to increase both the quantity and diversity of the dataset. For image datasets, various augmentation methods are available, including image scaling, random cropping, random flipping, color dithering, random erasing, Mixup, CutMix, Mosaic, and others to enhance data diversity [12]. Among these, Mosaic data augmentation is one of the most commonly used techniques for defect detection in insulation pull rod images. In fact, the Mosaic data augmentation method can improve data utilization efficiency, enhancing the diversity and robustness of the dataset, especially when the dataset is small. It has been widely applied in the processing of various image or video datasets [13,14].

However, this data augmentation method has some drawbacks. The generated images tend to have uniform sizes. Since there is an upper limit on the number of pixels in the images used for training, this results in a pixel limit for the images produced by Mosaic data augmentation. Consequently, the clarity of the original images is inevitably reduced, and the image size is decreased. Defect sizes in insulation pull rods vary, with defects such as bubbles and impurities being smaller in size. After applying Mosaic data augmentation, many labeled defects have a size smaller than 3 pixels, leading to poor correspondence between anchor boxes and the defect locations, which significantly hampers the accuracy of the training.

As for the typical defects observed in GIS, each individual defect conveys unique features and contours. White spots, scratches, and cracks are relatively larger in size and have more samples. The size of bubble defects is smaller, with a typical diameter of around 5 mm [15]. Impurity defects, on the other hand, vary greatly in size due to the wide variety of impurities, which include metal particles, hair, threads, and other contaminants [11]. Bubbles and metal impurities have very small sizes, with diameters ranging from 1 mm to 5 mm, and occupy a small proportion of the image in insulation pull rod pictures. Most image processing methods are designed for targets that occupy a relatively larger portion of the image, so when performing data augmentation, it is difficult to specifically enhance these small defects. In fact, the small size of bubbles and impurities may even lead to semantic loss in the image. Semantic loss refers to the degradation of small target features caused by downsampling or excessive enhancement in data augmentation.

In addition, due to the uneven distribution of defects in the actual production of pull rods, the proportion of defects such as white spots is much higher than that of defects such as bubbles. The proportion of imbalanced targets in the original data will be further imbalanced after expansion, resulting in a significant decrease in recognition performance. Kim et al. [16] indicated that the dataset significantly affects the accuracy of object recognition. Issues such as a small dataset size and an imbalanced distribution of different object types can lead to a decline in the model’s recognition performance. Khan et al. [17] proposed that data imbalance is a common problem in many practical applications, where the number of samples in most classes far exceeds that in a few classes. This imbalance can cause the classifier to lean towards the majority class, thereby affecting the recognition performance of the minority class. Therefore, when processing dataset augmentation, it is also necessary to consider the proportion balance of various targets.

Additionally, hair-like impurities are long in length but extremely narrow in width, with complex shapes. When labeling bounding boxes, a large portion of the area in the box is invalid. Therefore, the expanded areas are mostly invalid during data augmentation, which may result in distortion of semantic information.

To address the faced challenges, this study proposes a data preprocessing method that combines Mosaic data augmentation with in-image copy–pasting to enhance the defect features of insulation pull rods. Additionally, the bounding boxes for hair-like impurities are corrected, enabling the diverse shapes of these impurities to be standardized into two forms: diagonal and arc-shaped, thus improving detection accuracy for hair-like impurities. The YOLOv5s model, trained on the augmented dataset using this method, is employed to recognize defects in insulation pull rod images. The results show that, compared to using only Mosaic data augmentation, the model trained on the dataset expanded with this method and improved its mAP@0.5:0.95 from 0.618 to 0.789.

## 2. YOLOv5s Network Architecture and Procedure

### 2.1. Overview of YOLOv5s Network

**Figure 1:**
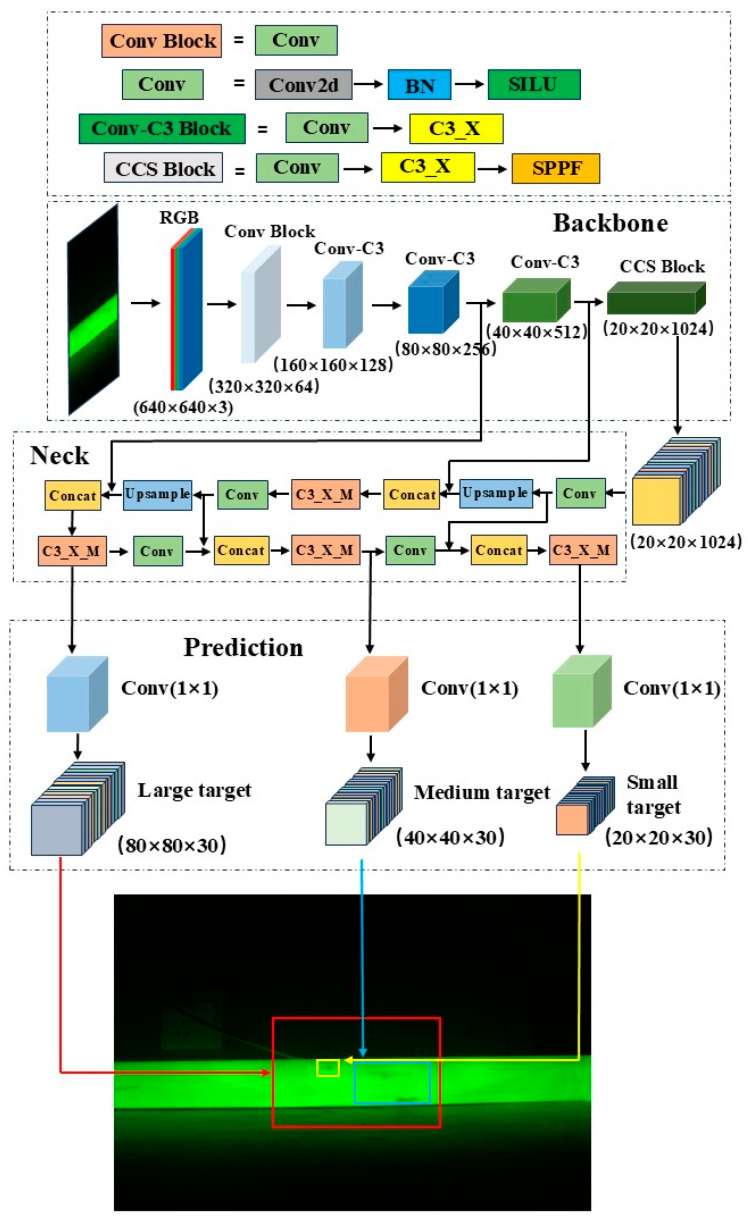
Structural diagram of defect detection model of insulating tie rod based on YOLOv5s.

In this study, the YOLOv5 model is used for defect detection, as it is a classic object detection model. Compared to other traditional object detection models, such as SSD and Faster R-CNN, YOLOv5 offers advantages, including fast detection speed, high accuracy, ease of training, and lightweight architecture [18,19]. YOLOv5 is also tailored for different application scenarios and is divided into four variants based on depth and feature map width: YOLOv5s, YOLOv5m, YOLOv5l, and YOLOv5x. Among these, the YOLOv5s model has the smallest depth and feature map width, while also providing faster frame rates and inference speeds [20]. Given that insulation pull rod defect detection requires both high accuracy and fast detection speed, the YOLOv5s model is the most suitable for this task. The YOLOv5s model consists of three main components: the backbone network which is responsible for feature extraction; the neck network which is responsible for feature fusion; and the prediction network. The procedure is shown in Figure 1.

The default training procedure for the YOLOv5s network is as follows: First, the input image is processed by resizing it to the preset dimensions of the network. In this study, the default size used is 640 × 640 pixels. The resized image is then split into its three RGB channels, and each pixel’s information is normalized to a floating-point number in the range of 0 to 1. Next, based on the requirements, the model can optionally use the default Mosaic data augmentation to expand the dataset, enhancing its robustness and diversity. Additionally, during the dataset preprocessing phase, the model calculates adaptive anchor boxes based on the pre-labeled bounding boxes in the dataset in order to determine the optimal anchor boxes for that particular dataset. Once the preprocessing steps are completed, the model begins several epochs of training. In each training epoch, the YOLO algorithm uses the original anchor boxes as references, calculates the differences between the predicted anchor boxes and the actual target locations, and updates the weight matrix using backpropagation. After training is finished, the model outputs the weight matrix corresponding to the best-performing round, which is then used for object detection.

### 2.2. Three Parts of YOLOv5s Network

#### 2.2.1. Backbone Feature Extraction Network

The main task of the backbone network is to extract features from images, converting the input raw image into multi-level feature maps. This part consists of the Conv module, C3_X module, and SPPF (Spatial Pyramid Pooling Fusion) module. The Conv module includes convolutional layers, batch normalization (BN) layers, and activation functions. The formula for the BN layer is shown in Equation (1), where the subscript *k* represents the *k*-th dimension of the sample, *x_k_* and *y_k_* represent the input and output samples of the BN layer, respectively; *β_k_* and *γ_k_* represent the shift and scale parameters, which are not hyperparameters but are learned through the network; and *μ_k_* and *σ_k_* represent the mean and standard deviation of the input samples [21].

(1) $$y_{k}={\;\gamma }_{k}\;\frac{x_{k}-\mu_{k}}{\sqrt{\;\sigma_{k}^{2}+\epsilon }}+\beta_{k}$$

The convolutional layers extract local features from the image, the BN layers normalize the extracted features, and the activation functions provide the network with nonlinear properties. The C3_X module stacks multiple convolutional operations with residual connections to efficiently extract multi-level information from the input features while reducing information loss during the feature extraction process. The SPPF module is introduced at the end of the backbone network to apply pooling operations of different scales to the convolutional feature maps, then fuses the pooled results to produce output feature maps with a consistent size, which can effectively expand the receptive field of the feature maps [22].

#### 2.2.2. Neck Feature Fusion Network

The neck network adopts a structure combining FPN (Feature Pyramid Network) and PAN (Path Aggregation Network) [23]. FPN uses a top-down approach to upsample the final feature map from the backbone network and fuses it with feature maps of the corresponding scales from earlier layers, enriching the feature representation. After repeating this process, the resulting fused feature maps have dimensions of 40 × 40 × 512 and 80 × 80 × 256. PAN uses a bottom-up strategy to process the lower-level feature maps of FPN through convolution operations, then fuses the results with feature maps of the same scale from FPN. This process is repeated until it reaches the top layer of FPN, producing fused feature maps of 40 × 40 × 256 and 20 × 20 × 512. FPN propagates high-level semantic information downwards, while PAN propagates positional information upwards. Together, they perform feature aggregation at different detection layers.

#### 2.2.3. Prediction Network

The YOLOv5 model’s loss function is divided into three parts: confidence, classification, and localization loss functions. The training process is essentially the process of minimizing the loss function. The localization loss function uses the GIOU loss function [24].

The formula for the YOLOv5 confidence loss function is shown in Equation (2) [25]. Here, *o* represents the true confidence value, which is 1 when sample *i* is a positive sample and 0 otherwise; $\hat{c_{i}}$ represents the predicted confidence value, indicating the probability that the model predicts sample *i* as a positive sample; and *N* is the total number of positive and negative samples.

(2) $$L_{\mathrm{conf}}\left(o,c\right)=-\frac{\sum_{i}\left(o_{i}\ln\left(\hat{c_{i}}\right)\;+\left(1-o_{i}\right)\ln\left(1-\hat{c_{i}}\right)\right)}{N},\;\;\hat{c_{i}}=\mathrm{Sigmoid}\left(c_{i}\right)$$

The formula for the YOLOv5 localization loss function is shown in Equation (3). In this formula, (*t_x_*, *t_y_*, *t_w_*_,_ *t_h_*) represents the predicted offset of sample *i* relative to the anchor box, where the coordinate values are not processed by the Sigmoid activation function; $\hat{l_{i}}$ represents the predicted offset of sample *i* after being processed by the Sigmoid function; ($g_{i}^{x}$, $g_{i}^{y}$_,_ $g_{i}^{w}$_,_ $g_{i}^{h}$) represents the true center coordinates and width or height of sample *i*; ($c_{i}^{x}$, $c_{i}^{y}$_,_ $p_{i}^{w}$_,_ $p_{i}^{h}$) represents the center coordinates and width or height of the anchor box *i*; and $\hat{g_{i}}$ represents the true offset of sample *i* relative to the anchor box.

(3) $$L_{\mathrm{loc}}\left(l,g\right)=\frac{\sum_{i\in \mathrm{pos}}\;\sum_{m\in \left\{x,y,w,h\right\}\;}L_{\mathrm{GIoU}\;}\left(\;{\hat{l_{i}}}^{m}-{\hat{g_{i}}}^{m}\right)}{N_{\mathrm{pos}}}$$

The prediction network outputs three feature maps of different scales to detect large, medium, and small objects. YOLOv5s divides the 640 × 640 × 3 input image into *N* × *N* grid cells and predicts information for each cell, including the bounding box center coordinates, width, height, classification probabilities, and confidence scores. The bounding box coordinates and size represent the precise position and size of the predicted object, while the confidence score indicates the likelihood that the object exists in the grid cell, with higher values suggesting a higher probability of the object’s presence. The classification probabilities determine the classification information of the predicted object.

**Figure 2:**
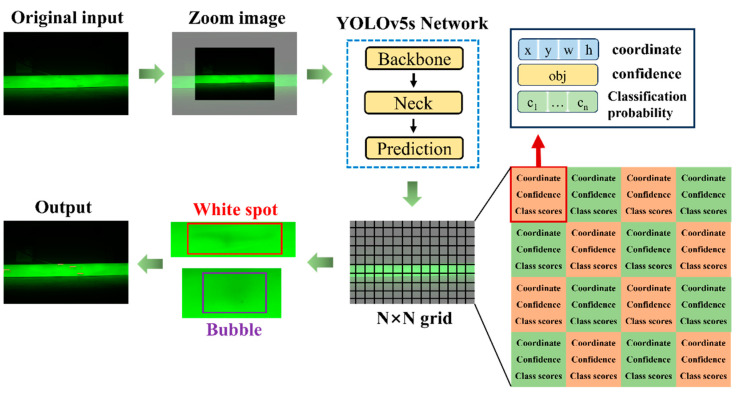
Detection process of YOLOv5s network.

First, the confidence score of each predicted bounding box is compared with a set threshold. Predictions with confidence scores below the threshold are discarded, while those above the threshold are considered to contain targets, with the position and size of the detected targets obtained. Next, the Non-Maximum Suppression (NMS) algorithm is applied to filter duplicate bounding boxes for the same target [26]. Finally, the target’s index and category are determined based on the highest classification probability. The detection process of YOLOv5s network is shown in Figure 2.

## 3. Two Preprocessing Methods for Insulation Pull Rod Defect Dataset

### 3.1. Copy–Paste Data Augmentation

For small-sized bubble and impurity defects, directly applying Mosaic data augmentation leads to a significant reduction in the size of the defects in the generated images, which in turn results in a marked decrease in training performance. In this study, we use the copy–paste method for augmenting the defect dataset. The approach involves copying a portion of the image from within the bounding box of the original image and pasting it randomly multiple times within the same image, thereby increasing the number of corresponding targets and enhancing the diversity of the dataset. In the original image, the insulation pull rod typically occupies only a central portion, while the top and bottom areas are mostly background, so the pasted regions are likely to fall into the previously inactive areas, ensuring that the defect regions are not obscured.

This data augmentation method is applied only to bubble and impurity defects. For each image, if there are multiple bubble or impurity defects, the copy–paste operation is applied to each defect individually. Other types of defects are left untouched. The same original image can be augmented into multiple processed images, effectively increasing the number of bubble and impurity defect samples.

**Figure 3:**
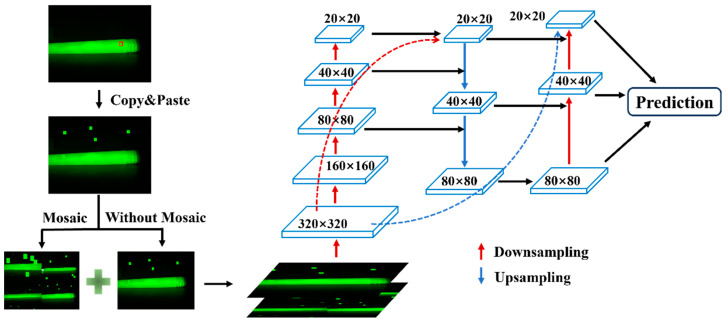
Illustration of the Copy–Paste Data Augmentation Method. The area marked with a red box indicates the defect target area.

After performing copy–paste on the image, the resulting images are combined with the original images for Mosaic data augmentation. The Mosaic-augmented images are then mixed with images that only underwent copy–paste augmentation, forming the training dataset for the network. In the neck network, FPN upsamples from top to bottom, while PAN applies convolution to the lower-level feature maps of FPN in a bottom-up manner, and then fuses the convolution results with feature maps of the same scale from FPN. This generates three fused feature maps of sizes 20 × 20 × 1024, 40 × 40 × 512, and 80 × 80 × 256. The procedure of Copy–Paste Data Augmentation Method is shown in Figure 3.

This method is particularly effective for small-sized impurity and bubble defects. After data augmentation, it preserves the large features in the dataset while also retaining the small-sized features introduced by Mosaic. In other words, it adds large and medium-sized defects of the corresponding types to the dataset, significantly increasing the number of these two types of defects, and leading to a more balanced distribution of different defect types in the dataset.

### 3.2. Correcting Bounding Boxes for Hair-like Impurities

Hair and threads are typical impurities that may occasionally fall into the raw materials during the production of insulation pull rods. These impurities typically feature very fine widths, and long lengths, and are prone to bending into various shapes. During training, YOLOv5s uses a horizontal rectangular bounding box format, which cannot rotate, making it prone to labeling a large number of irrelevant areas when annotating hair-like impurities. This significantly deteriorates the training results.

To address this issue, this study proposes a correction to the annotation method for hair-like impurities. Instead of annotating a single continuous hair strand, the hair is divided into multiple segments. These segments take two main shapes: diagonal and arc-shaped. A diagonal-shaped segment extends from one vertex of the bounding box to the opposite vertex, while an arc-shaped segment extends from one vertex to another vertex on the same side of the box. This method simplifies the diverse shapes of hair-like impurities into two more regular forms, making them more typical and easier to identify. Moreover, during detection, even if a small part of the hair impurity is not detected, the presence of any detected region is sufficient to consider the defect recognized.

The principles of hair impurity bounding box correction include the following: (1) The angle difference between diagonal hair impurities and the diagonal body should not exceed ±15 degrees; (2) The radius of the annotation box for arched hair impurities should not exceed 90 degrees of the corresponding curvature circle; and (3) To avoid the annotation box being too small, the number of segments should not exceed six every 360 degrees.

**Figure 4:**
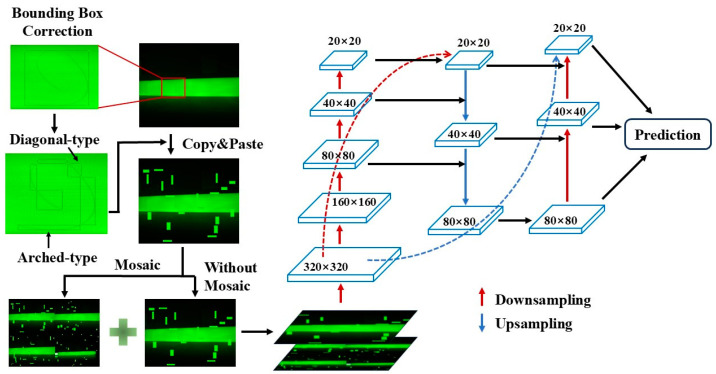
Correction to bounding boxes of hair-like impurities.

The process begins by applying the above annotation method to the hair-like impurities in the original defect images of the insulation pull rods. Then, the annotated hair-like impurities are subject to the copy–paste augmentation, and the augmented images are combined with the original images for Mosaic data augmentation. These Mosaic-augmented images, along with the images that only underwent copy–paste augmentation, are then mixed to form the training dataset for the network. After passing through the FPN and PAN feature extraction layers in the YOLOv5s neck network, three fused feature maps with sizes 20 × 20 × 1024, 40 × 40 × 512 and 80 × 80 × 256 are generated. This approach operates solely on hair-like impurities based on the copy–paste augmentation, without making any additional adjustments for other types of defects. The procedure of bounding boxes of hair-like impurities bounding boxes is shown in Figure 4.

## 4. Experiment Results and Analysis

### 4.1. Overview

In this study, the YOLOv5s model was trained on multiple datasets to compare its detection performance and validate the effectiveness of the preprocessing methods described earlier. A total of seven datasets were set up, named Dataset 1 through Dataset 7. Each dataset contains 8000 images, with the detection targets being five types of insulation pull rod defects: white spots, impurities, bubbles, cracks, and scratches.

However, in the original dataset, the proportion of various types of defects is very unbalanced, and the specific quantities and proportions of the five types of defects are shown in Table 1.

Before data augmentation, there were a total of 397 original images, of which 350 images were taken as the expanded basis for the training set, including five types of defects. The remaining 47 images will be kept as the validation set. After data augmentation, the original 350 images were expanded to 8000 images. Each target type defect was copied and pasted five times.

As shown in Table 2, these datasets were used to evaluate the performance of the model with different preprocessing techniques.

To evaluate the effectiveness of the data preprocessing methods, mAP@0.5 and mAP@0.5:0.95 are used as evaluation metrics for detection accuracy, where mAP (mean average precision) represents the average detection accuracy across all target types, and AP (average precision) represents the average detection accuracy for a specific target type. The values of mAP and AP can be computed using Equations (4) and (5):

(4) $$\mathrm{mAP}=\frac{1}{n}\sum AP_{i}$$

(5) $$\mathrm{AP}=\int_{0}^{1}P(R)dR$$

In Equation (5), the average precision is the area under the P-R (Precision-Recall) curve, where P(Precision) and R(Recall) are defined by Equations (6) and (7):

(6) $$P=\frac{T_{P}}{T_{P}+F_{P}}$$

(7) $$R=\frac{T_{P}}{T_{P}+F_{N}}$$

T_P_ (True Positive) refers to cases where the actual label is positive, and the prediction is also positive. T_N_ (True Negative) refers to cases where the actual label is negative, and the prediction is also negative. F_P_ (False Positive) refers to cases where the actual label is negative, but the prediction is positive. F_N_ (False Negative) refers to cases where the actual label is positive, but the prediction is negative.

F1 score is another important parameter for evaluating the effectiveness of a model, which can be used to comprehensively assess the balance between P and R. The value of F1 score can be computed using Equation (8):

(8) $$F1\;\mathrm{Score}=2\cdot \frac{\mathrm{PR}}{P+R}$$

Based on these definitions, precision reflects the model’s ability to correctly detect positive instances, and recall reflects the proportion of actual positive instances correctly predicted by the model. In this study, the values of mAP@0.5, mAP@0.5:0.95, P, R and F1 score for each defect type are used as the evaluation criteria. Specifically, mAP@0.5 refers to the mAP value when the Intersection over Union (IoU) threshold is set to 0.5, while mAP@0.5:0.95 refers to the average mAP value when the IoU threshold is varied from 0.05 to 0.95 in steps of 0.05.

The Python version used in this study is 3.9.19, and the PyTorch version is 2.3.1.

### 4.2. Validation of the Effectiveness of Two Methods

#### 4.2.1. Validation of the Effectiveness of the Copy–Paste Method

In this study, seven training datasets were used. By training with Dataset 1, Dataset 2, and Dataset 5, in which no correction to the bounding boxes of hair-like impurities was applied, we can compare whether combining the copy–paste method with Mosaic data augmentation improves the defect detection performance of insulation pull rods. The training was conducted using the same parameters; the starting weight file was the default YOLOv5s model weight file, and the number of training epochs was set to 100. Since the datasets had already undergone data augmentation, no further augmentation was applied during training. The training results are shown in Table 3.

Table 3 presents the training results for three datasets, listing the number of annotated defects of each type after data augmentation, along with the corresponding mAP@0.5 and mAP@0.5:0.95 values. From the table, it can be observed that when all images in the dataset undergo Mosaic data augmentation, the total number of defect samples is the highest, but the distribution is uneven. This is because, in the original data, the number of white spots and impurities is already high; therefore, after augmentation, these two defect types account for 75% of the total samples. Although the number of crack and scratch samples is relatively small, these defects are larger in size and have more distinct features, leading to better detection results. In contrast, the bubble defects, which originally constituted only 4.8% of the dataset, are smaller in size, leading to poorer training results. The detection performance for impurities is also relatively poor.

As the number of augmented images using the copy–paste method increases, the total number of samples decreases, but the numbers of bubble and impurity defect samples decrease less or even increase. The distribution of defects becomes more balanced. It can be observed that the detection performance for bubble defects has significantly improved, while the detection of the other defect types has not noticeably declined. Although the recognition accuracy for white spots, cracks, and scratches in Datasets 2 and 4 is slightly lower than in Dataset 1, this decline is closely related to the reduction in number of samples. In future work, increasing the sample size for these two defect types can help improve their recognition performance.

Based on the Precision, Recall, and F1 score metrics, the precision values of all three datasets are relatively high; however, in Dataset 1, the recall values for bubble and impurity defects are comparatively low. This indicates that in the dataset trained using default Mosaic data augmentation, most of the identified bubble and impurity defects are correctly recognized, but a significant proportion of defects are still missed. In contrast, after applying in-image copy–paste augmentation for bubble and impurity defects, as the number of augmented images increases, the recall for bubbles significantly improves, while the increase in recall for impurities is relatively modest. Consequently, the F1 scores for both bubbles and impurities improve with the increasing number of copy–paste augmented images. Specifically, the F1 score for bubbles increases from 0.585 to 0.913 (Table 3, Dataset 1 and Dataset 5, “Bubble” column), which is comparable to the F1 scores of other defect types; however, the F1 score for impurities remains significantly lower at only 0.793—more than 0.1 below that of other types (Table 3, Dataset 1 and Dataset 5, “Impurity” column).

This suggests that the enhancement in impurity detection is limited, indicating that the proposed method is less effective for irregular defects like impurities, and that alternative approaches are needed to further improve impurity detection performance.

#### 4.2.2. Validation of the Effectiveness of Hair-like Impurity Bounding Boxes Correction

By comparing Datasets 2, 3, and 4, and separately comparing Datasets 5, 6, and 7, we can evaluate whether the bounding box correction of hair-like impurities improves the defect detection performance of the insulation pull rod. The same training parameters were used, with the starting weight file being the default YOLOv5s model weights. The number of training epochs was set to 100, and since each dataset had already undergone data augmentation, no further augmentation was applied during training. The training results are shown in Table 4 and Table 5.

From the results shown in the tables, it is evident that, under otherwise identical conditions, correcting the bounding boxes for hair-like impurities can significantly improve the recognition accuracy of impurity-related defects.

For Dataset 2, after correcting the bounding boxes for hair-like impurities and applying the copy–paste augmentation method, the mAP@0.5:0.95 for impurity defects increased by 8.3%. If the images with corrected bounding boxes are further subjected to Mosaic data augmentation, combined with copy–paste augmentation, the mAP@0.5:0.95 for impurity defects increased by 23.2%, with the mAP@0.5 surpassing 90%.

For Dataset 5, after correcting the bounding boxes for hair-like impurities and applying only the copy–paste augmentation, the mAP@0.5:0.95 for impurity defects increased by 3.3%. When the corrected images were further augmented using Mosaic data augmentation combined with copy–paste method, the mAP@0.5:0.95 for impurity defects increased by 13.8%, and the mAP@0.5 approached 95%.

Based on the Precision, Recall, and F1 score metrics, under otherwise identical conditions, correcting the bounding boxes for hair-like impurities can effectively improve the recall for impurity defects, thereby also enhancing their F1 score. For Dataset 2, the F1 score for impurity defects increased from 0.678 to 0.889 (Table 4, Dataset 2 and Dataset 4, “Impurity” column); for Dataset 5, it increased from 0.793 to 0.913 (Table 5, Dataset 5 and Dataset 7, “Impurity” column). Meanwhile, the Precision, Recall, and F1 scores for other defect types were not significantly affected. Therefore, it can be concluded that correcting the bounding boxes for hair-like impurities enables the model to more accurately detect these impurities, ultimately improving the detection rate of impurity defects.

**Figure 5:**
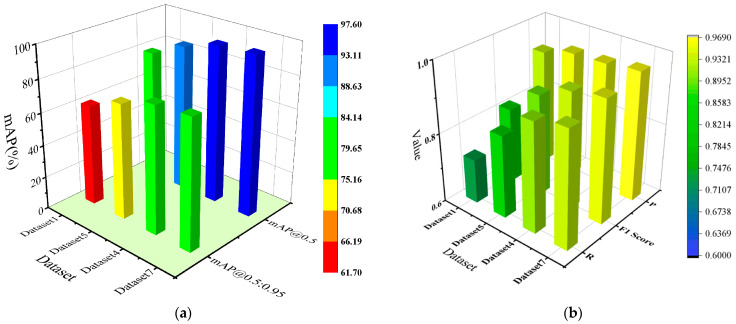
Comparison of mAP@0.5:0.95 Performance and P/R/F1 score performance across different data augmentation methods. (**a**) Comparison of mAP@0.5:0.95 performance across different data augmentation methods; (**b**) Comparison of P/R/F1 score performance across different data augmentation methods.

The results show that correcting the bounding boxes for hair-like impurities, followed by Mosaic data augmentation and combined with copy–paste, performs significantly better than applying only copy–paste after the bounding box correction, while still using the original images for Mosaic data augmentation. Moreover, this method not only improves the recognition accuracy of impurity defects but also enhances the detection of other types of defects to a certain extent. For Dataset 2, the overall mAP@0.5:0.95 for all defect types increased by 13.6%, Precision for all defect types increased by 0.5%, Recall for all defect types increased by 14.2%, and the F1 Score for all defect types increased by 8.2% (Table 4, Dataset 2 and Dataset 4, “All” column). For Dataset 5, the overall mAP@0.5:0.95 for all defect types increased by 8.2%, Precision for all defect types increased by 1.3%, Recall for all defect types increased by 9.7%, and F1 Score for all defect types increased by 5.9% (Table 5, Dataset 5 and Dataset 7, “All” column). The comparison of mAP@0.5:0.95 performance and P/R/F1 score performance cross different data augmentation methods, as Figure 5 shows.

Figure 5 shows a comprehensive comparison of the best-performing datasets in each group. It can be observed that correcting the bounding boxes for hair-like impurities, followed by Mosaic data augmentation combined with copy–paste, resulted in a significant improvement in detection performance compared to the model trained solely with Mosaic data augmentation. The overall mAP@0.5:0.95 for all defect types increased from 61.8% to 78.9%, and a corresponding improvement of 17.1% is observed as well. The overall F1 score for all defect types increased from 0.816 to 0.951, and a corresponding improvement of 13.5% is observed as well (Table 3 and Table 5, Dataset 1 and Dataset 7, “All” column).

The model complexity during training is 7,033,114 parameters, 16.0 GFLOPs. The inference time and GPU memory consumption are listed in Table 6.

It can be found that, after copying and pasting in the image and modifying the hair impurity bounding box, the inference time is not affected much, and the GPU memory consumption is almost unchanged.

### 4.3. Comparison of Detection Results on Real Photos

**Figure 6:**
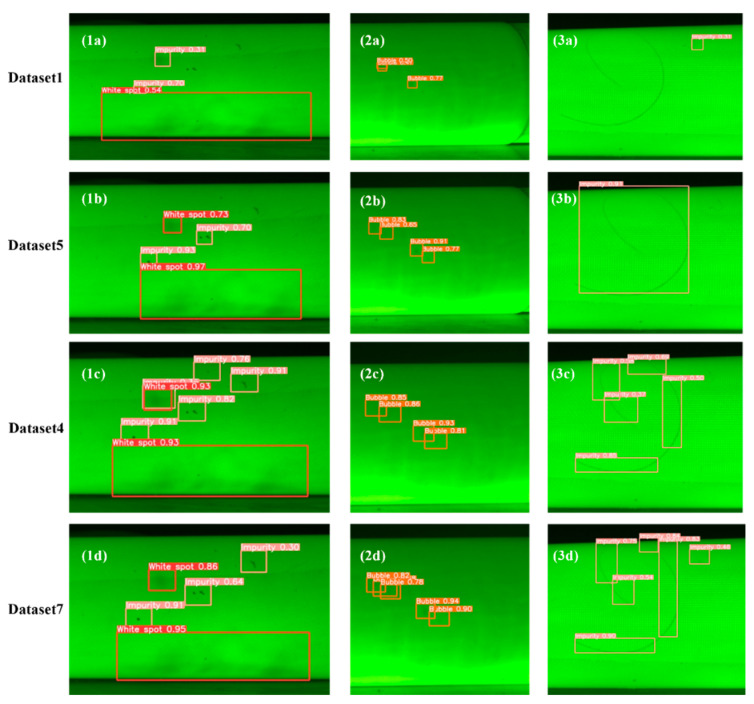
Comparison of detection results on real photos: (**1a**) Pull Rod 1, Dataset 1; (**1b**) Pull Rod 1, Dataset 5; (**1c**) Pull Rod 1, Dataset 4; (**1d**) Pull Rod 1, Dataset 7; (**2a**) Pull Rod, Dataset 1; (**2b**) Pull Rod 2, Dataset 5; (**2c**) Pull Rod 2, Dataset 4; (**2d**) Pull Rod 2, Dataset 7; (**3a**) Pull Rod 3, Dataset 1; (**3b**) Pull Rod 3, Dataset 5; (**3c**) Pull Rod 3, Dataset 4; (**3d**) Pull Rod 3, Dataset 7.

To further evaluate the effectiveness of the two data preprocessing methods in improving the defect detection accuracy of insulation rods, Figure 6 presents the detection results of models trained on different datasets using the same set of images. Three test images were selected for comparison. The first image contains multiple defects of white spots and impurities; the second image contains multiple bubble defects; and the third image includes both a large and small hair-like impurity. The models trained on datasets 1, 5, 4, and 7 were named A, B, C, and D, respectively.

From the detection results of the first image, it can be observed that models A and B failed to detect all the impurity defects, with model A having noticeably lower detection confidence. Model C detected all the defects but made a false positive, misclassifying a white spot as both a white spot and an impurity. Model D did not make any false positives, but missed a small impurity defect, and its confidence was slightly lower than that of Model C. In the second image, Model A failed to detect all the bubble defects, while Models B, C, and D successfully detected all bubble defects, with Models C and D showing similar confidence, both higher than Model B. In the third image, Model A completely failed to detect the larger hair impurity, while Models B and C missed the smaller hair-like impurity. Model D successfully detected both hair-like impurities, with better bounding box coverage and higher confidence than Model C.

Comparing these results, it is clear that using only Mosaic data augmentation leads to poorer detection performance. In contrast, combining image copy-pasting with Mosaic augmentation significantly improves the model’s detection accuracy. Additionally, correcting the bounding boxes for hair-like impurities enhances the model’s accuracy in detecting this type of defect. However, the ratio of image copy-pasting to Mosaic-augmented images should not be excessively high, as an overly large number of copy–pasted images may not significantly improve the detection accuracy and could even lead to a decrease in performance.

## 5. Conclusions

The traditional inspection methods used for insulation rods after production are often subjective, time-consuming, and labor-intensive, which may result in missed detections or errors. This study proposes using a YOLOv5s-based image recognition system for defect detection in insulation pull rods and introduces a combined data augmentation method involving in-image copy & paste and Mosaic augmentation. Additionally, a framework for correcting bounding boxes for hair-like impurities is proposed to improve the model’s accuracy in detecting defects. Based on the above work, the following conclusions can be drawn:

(1) Comparing with traditional Mosaic data augmentation, the in-image copy–paste technique significantly improves the detection accuracy of small bubble defects. This approach increases the mAP@0.5 for bubble defects from 0.531 to 0.904, increases the mAP@0.5:0.95 from 0.329 to 0.766 and increases the F1 score for bubbles from 0.585 to 0.913 (Table 3, Dataset 1 and Dataset 5, “Bubble” column) It also raises the overall mAP@0.5 from 0.797 to 0.897, raises the overall mAP@0.5:0.95 from 0.618 to 0.707 and raises the overall F1 score from 0.816 to 0.892 (Table 3, Dataset 1 and Dataset 5, “All” column).
(2) Correcting the bounding boxes for typical hair-like impurities improves the detection accuracy of these defects. Standardizing the diverse shapes of hair-like impurities into two regular forms-arched-type and diagonal-type, combined with the in-image copy–paste and Mosaic data augmentation approach, improves the detection of impurity defects. The mAP@0.5 for hair-like impurities increases from 0.777 to 0.946, mAP@0.5:0.95 rises from 0.593 to 0.731, and the F1 score rises from 0.793 to 0.913(Table 5, Dataset 5 and Dataset 7, “Impurity” column). Meanwhile, the overall mAP@0.5 improves from 0.897 to 0.976, overall mAP@0.5:0.95 increases from 0.707 to 0.789, and the overall F1 score rises from 0.892 to 0.951(Table 5, Dataset 5 and Dataset 7, “All” column).
(3) The proportion of images generated through the in-image copy–paste method should not be too high, as an excessive number of such images may negatively impact detection accuracy.

There are still several promising directions remaining for future work. Introducing Generative Adversarial Networks (GANs) to synthesize high-resolution defect samples and further alleviate data imbalance is a feasible option [27]. Meanwhile, investigating feature fusion enhancements or hybrid detection approaches will also benefit the improvement of the model.

## Figures and Tables

**Table 1:** The specific quantity and proportion of the five types of defects.

Defect Type	Quantity	Proportion
White spot	483	58.2%
Impurity	138	16.6%
Bubble	40	4.8%
Crack	61	7.4%
Scratch	108	13.0%

**Table 2:** Composition of datasets and preprocessing methods.

Dataset		Copy–Paste	Hair-like Impurities Bounding Box Correction	Mosaic with Hair-like Impurities Bounding Box Correction	Mosaic without Hair-like Impurities Bounding Box Correction
Dataset 1	Adopted/Not Adopted	×	×	×	√
	Quantity	0	**/**	0	8000
Dataset 2	Adopted/Not Adopted	√	×	×	√
	Quantity	2000	/	0	6000
Dataset 3	Adopted/Not Adopted	√	√	×	√
	Quantity	2000	/	0	6000
Dataset 4	Adopted/Not Adopted	√	√	√	×
	Quantity	2000	/	6000	0
Dataset 5	Adopted/Not Adopted	√	×	×	√
	Quantity	4000	/	0	4000
Dataset 6	Adopted/Not Adopted	√	√	×	√
	Quantity	4000	/	0	4000
Dataset 7	Adopted/Not Adopted	√	√	√	×
	Quantity	4000	/	4000	0

**Table 3:** Comparison of training results for different data augmentation methods.

Dataset		White Spot	Impurity	Bubble	Crack	Scratch	All
Dataset 1	Number of instances	38,855	11,234	3186	4769	8714	66,758
	mAP@0.5	0.958	0.664	0.531	0.907	0.928	0.797
	mAP@0.5:0.95	0.796	0.437	0.329	0.752	0.775	0.618
	Precision	0.948	0.894	0.888	0.966	0.962	0.931
	Recall	0.913	0.555	0.436	0.855	0.877	0.727
	F1 Score	0.930	0.685	0.585	0.907	0.918	0.816
Dataset 2	Number of instances	30,021	10,402	4740	3520	6641	55,324
	mAP@0.5	0.955	0.659	0.77	0.904	0.933	0.844
	mAP@0.5:0.95	0.774	0.428	0.531	0.723	0.746	0.641
	Precision	0.948	0.921	0.971	0.959	0.969	0.954
	Recall	0.907	0.536	0.7	0.841	0.873	0.771
	F1 Score	0.927	0.678	0.814	0.896	0.918	0.853
Dataset 5	Number of instances	20,858	9666	6424	2340	4494	43,782
	mAP@0.5	0.95	0.777	0.904	0.91	0.943	0.897
	mAP@0.5:0.95	0.738	0.593	0.766	0.702	0.737	0.707
	Precision	0.954	0.924	0.981	0.956	0.966	0.956
	Recall	0.903	0.695	0.853	0.853	0.874	0.836
	F1 Score	0.928	0.793	0.913	0.902	0.918	0.892

**Table 4:** Comparison of training results for datasets with and without hair-like impurity bounding box correction (Datasets 2, 3 and 4).

Dataset		White Spot	Impurity	Bubble	Crack	Scratch	All
Dataset 2	Number of instances	30,021	10,402	4740	3520	6641	55,324
	mAP@0.5	0.955	0.659	0.77	0.904	0.933	0.844
	mAP@0.5:0.95	0.774	0.428	0.531	0.723	0.746	0.641
	Precision	0.948	0.921	0.971	0.959	0.969	0.954
	Recall	0.907	0.536	0.7	0.841	0.873	0.771
	F1 Score	0.927	0.678	0.814	0.896	0.918	0.853
Dataset 3	Number of instances	29,684	11,004	4728	3520	6632	55,748
	mAP@0.5	0.956	0.726	0.787	0.913	0.934	0.863
	mAP@0.5:0.95	0.775	0.511	0.635	0.737	0.765	0.684
	Precision	0.953	0.917	0.943	0.964	0.971	0.95
	Recall	0.909	0.626	0.717	0.858	0.871	0.796
	F1 Score	0.930	0.744	0.815	0.908	0.918	0.866
Dataset 4	Number of instances	29,389	12,245	4966	3591	6739	57,380
	mAP@0.5	0.991	0.922	0.925	0.992	0.991	0.964
	mAP@0.5:0.95	0.817	0.66	0.733	0.835	0.837	0.777
	Precision	0.969	0.931	0.937	0.978	0.981	0.959
	Recall	0.964	0.851	0.833	0.957	0.962	0.913
	F1 Score	0.966	0.889	0.882	0.967	0.971	0.935

**Table 5:** Comparison of training results for datasets with and without hair-like impurity bounding box correction (Datasets 5, 6, and 7).

Dataset		White Spot	Impurity	Bubble	Crack	Scratch	All
Dataset 5	Number of instances	20,858	9666	6424	2340	4494	43,782
	mAP@0.5	0.95	0.777	0.904	0.91	0.943	0.897
	mAP@0.5:0.95	0.738	0.593	0.766	0.702	0.737	0.707
	Precision	0.954	0.924	0.981	0.956	0.966	0.956
	Recall	0.903	0.695	0.853	0.853	0.874	0.836
	F1 Score	0.928	0.793	0.913	0.902	0.918	0.892
Dataset 6	Number of instances	20,661	10,830	6400	2340	4476	44,707
	mAP@0.5	0.95	0.805	0.899	0.91	0.939	0.9
	mAP@0.5:0.95	0.744	0.626	0.771	0.703	0.737	0.716
	Precision	0.967	0.941	0.987	0.97	0.979	0.969
	Recall	0.891	0.715	0.849	0.833	0.859	0.83
	F1 Score	0.927	0.813	0.913	0.896	0.915	0.894
Dataset 7	Number of instances	20,583	11,659	6565	2309	4522	45,638
	mAP@0.5	0.99	0.946	0.969	0.991	0.985	0.976
	mAP@0.5:0.95	0.798	0.731	0.816	0.799	0.804	0.789
	Precision	0.971	0.934	0.981	0.976	0.981	0.969
	Recall	0.958	0.893	0.919	0.946	0.948	0.933
	F1 Score	0.964	0.913	0.949	0.961	0.964	0.951

**Table 6:** The inference time and GPU memory consumption of each dataset.

Dataset	Inference Time (ms)	GPU Memory Consumption
Dataset 1	22.9	7.35 G
Dataset 2	23.0	7.34 G
Dataset 3	22.5	7.35 G
Dataset 4	25.1	7.35 G
Dataset 5	23.6	7.35 G
Dataset 6	23.4	7.35 G
Dataset 7	21.5	7.34 G

## Data Availability

Due to the nature of this research, study participants did not agree for their data to be shared publicly, so supporting data are not available.

## References

[1] Li C., Lin C., Chen G., Tu Y., Zhou Y., Li Q., Zhang B., He J. (2019). Field-Dependent Charging Phenomenon of HVDC Spacers Based on Dominant Charge Behaviors. Appl. Phys. Lett..

[2] Li C., Zhu Y., Hu J., Li Q., Zhang B., He J. (2020). Charge Cluster Triggers Unpredictable Insulation Surface Flashover in Pressurized SF6. J. Phys. D Appl. Phys..

[3] Li C., Yang Y., Xu G., Zhou Y., Jia M., Zhong S., Gao Y., Park C., Liu Q., Wang Y. (2022). Insulating Materials for Realising Carbon Neutrality: Opportunities, Remaining Issues and Challenges. High Voltage.

[4] Xing Y., Wang Z., Liu L., Xu Y., Yang Y., Liu S., Zhou F., He S., Li C. (2022). Defects and Failure Types of Solid Insulation in Gas-Insulated Switchgear: In Situ Study and Case Analysis. High Voltage.

[5] Feng Z., Guo L., Huang D., Li R. (2021). Electrical Insulator Defects Detection Method Based on YOLOv5. Proceedings of the 2021 IEEE 10th Data Driven Control and Learning Systems Conference (DDCLS).

[6] Liu L., Huang K., Bai Y., Zhang Q., Li Y. (2024). Real-Time Detection Model of Electrical Work Safety Belt Based on Lightweight Improved YOLOv5. J. Real-Time Image Process..

[7] Nguyen R. (2023). Object Detection with YOLOv5 for Electric Utility Asset Inspection Using UAVs. Master’s Thesis.

[8] Pérez-Aguilar D.A., Pérez-Aguilar J.M., Pérez-Aguilar A.P., Risco-Ramos R.H., Malpica-Rodríguez M.E. (2024). Electric Substation Inspection: YOLOv5 in Hotspot Detection Through Thermal Imaging. Ingenius.

[9] Li Y., Ni M., Lu Y. (2022). Insulator Defect Detection for Power Grid Based on Light Correction Enhancement and YOLOv5 Model. Energy Rep..

[10] Liang F., Zhao L., Ren Y., Wang S., To S., Abbas Z., Islam M.S. (2024). LAD-Net: A lightweight welding defect surface non-destructive detection algorithm based on the attention mechanism. Comput. Ind..

[11] Li C., Hua Y., Liu Y., Liu K., Zhang S. (2024). Intelligent Identification Method of Insulation Pull Rod Defects Based on Intactness-Aware Mosaic Data Augmentation and Fusion of YOLOv5s. High Voltage.

[12] Nisa U. (2024). Image Augmentation Approaches for Small and Tiny Object Detection in Aerial Images: A Review. Multimed. Tools Appl..

[13] Teterja D., Garcia Rodriguez J., Azorin-Lopez J., Sebastian-Gonzalez E., Nedic D., Lekovic D., Knezevic P., Drajic D., Vukobratovic D. (2024). A Video Mosaicing-Based Sensing Method for Chicken Behavior Recognition on Edge Computing Devices. Sensors.

[14] Li Z., Li Y., Li H., Deng L., Yan R. (2024). Surround Sensing Technique for Trucks Based on Multi-Features and Improved Yolov5 Algorithm. Sensors.

[15] Dai Y., Chen G., Zhang Y., Zhu Y., Wang Z., Li W., Wang C., Tu Y. (2024). Electric Field Distribution of Micro Surface Scratch Defects in GIS Insulation Pull Rods. Proceedings of the 2024 IEEE International Conference on High Voltage Engineering and Applications (ICHVE), Berlin, Germany, 18–22 August 2024.

[16] Kim J.-H., Kim N., Park Y.W., Won C.S. (2022). Object Detection and Classification Based on YOLO-V5 with Improved Maritime Dataset. J. Mar. Sci. Eng..

[17] Hasib K.M., Iqbal M.S., Shah F.M., Mahmud J.A., Popel M.H., Showrov M.I., Ahmed S., Rahman O. (2020). A Survey of Methods for Managing the Classification and Solution of Data Imbalance Problem. J. Comput. Sci..

[18] Redmon J., Divvala S., Girshick R., Farhadi A. (2016). You Only Look Once: Unified, Realtime Object Detection. Proceedings of the IEEE Conference on Computer Vision and Pattern Recognition.

[19] Bochkovskiy A., Wang C.Y., Liao H.Y.M. (2020). YOLOv4: Optimal Speed and Accuracy of Object Detection. arXiv.

[20] Wang X., Wang X., Hu C., Dai F., Xing J., Wang E., Du Z., Wang L., Guo W. (2022). Study on the Detection of Defoliation Effect of an Improved YOLOv5x Cotton. Agriculture.

[21] Ioffe S., Szegedy C. (2015). Batch Normalization: Accelerating Deep Network Training by Reducing Internal Covariate Shift. arXiv.

[22] He K., Zhang X., Ren S., Sun J. (2015). Spatial Pyramid Pooling in Deep Convolutional Networks for Visual Recognition. IEEE Trans. Pattern Anal. Mach. Intell..

[23] Liu S., Qi L., Qin H., Shi J., Jia J. (2018). Path Aggregation Network for Instance Segmentation. Proceedings of the IEEE Conference on Computer Vision and Pattern Recognition (CVPR).

[24] Rezatofighi H., Tsoi N., Gwak J.Y., Sadeghian A., Reid I., Savarese S. (2019). Generalized Intersection over Union: A Metric and a Loss for Bounding Box Regression. Proceedings of the IEEE/CVF Conference on Computer Vision and Pattern Recognition (CVPR).

[25] Redmon J., Farhadi A. (2018). YOLOv3: An Incremental Improvement. arXiv.

[26] Qiu S., Wen G., Deng Z., Liu J., Fan Y. (2018). Accurate Non-Maximum Suppression for Object Detection in High-Resolution Remote Sensing Images. Remote Sens. Lett..

[27] Rezazadeh N., Perfetto D., Polverino A., De Luca A., Lamanna G. (2024). Guided wave-driven machine learning for damage classification with limited dataset in aluminum panel. Struct. Health Monit..

